# The association between six month intra-dialytic resistance training and muscle strength or physical performance in patients with maintenance hemodialysis: a multicenter retrospective observational study

**DOI:** 10.1186/s12882-019-1375-1

**Published:** 2019-05-16

**Authors:** Yoshifumi Moriyama, Masahiko Hara, Sae Aratani, Hideaki Ishikawa, Kenichi Kono, Masatake Tamaki

**Affiliations:** 1 Department of Health Fitness Program, Nagoya Kyoritsu Hospital, Nagoya, Japan; 2 Department of Clinical Investigation, Japan Society of Clinical Research, Kita-ku Umeda 1-11-1000, Osaka Ekimae 4th Bldg 10F, Osaka, 530-0001 Japan; 3 Center for Community-based Healthcare Research and Education, Shimane University, Izumo, Japan; 4 Department of Nephrology, Nippon Medical School Graduate School of Medicine, Tokyo, Japan; 5 Department of Nephrology, Nagoya First Red Cross Hospital, Aichi, Japan; 6 Department of Physical Therapy, International University of Health and Welfare School of Health Science at Narita, Narita, Japan; 7 Department of Minimally Invasive Surgical and Medical Oncology, Fukushima Medical University, Fukushima, Japan

**Keywords:** Hemodialysis, Muscle strength, Physical performance, Quality of life, Resistance training

## Abstract

**Background:**

Reduced muscle strength and physical performance are prevalent in patients of maintenance hemodialysis (MHD), and deleterious changes in these parameters are associated with increased mortality.

**Methods:**

This retrospective observational study included 306 patients, who received a 6-month resistance exercise program during hemodialysis, three times per week on an outpatient basis. The training protocol consisted of two sets of 10 repetitions of knee extension, hip abduction, and hip flexion, using an elastic band in a sitting or supine position. Primary outcome measures included muscle strength, measured by percent knee extension muscle power to dry body weight (pKEMP-dBW), and physical performance, measured by short physical performance battery (SPPB). The adjusted mean differences in these variables during the 6 months were estimated using a multivariate linear regression model.

**Results:**

The mean age with standard deviation was 70 ± 11 years. One hundred and sixty patients (52.3%) were men and the dry weight was 55.6 ± 11.3 kg. Sarcopenia, defined as SPPB ≤8, was present in 21.4% patients. Their hemodialysis adequacy was acceptable, with a Kt/V of 1.65 ± 0.29, and their nutritional status was good, with a normalized protein catabolism rate of 0.89 ± 0.18 g/kg/day. During the 6 months, both pKEMP-dBW and SPPB showed a slight but significant increase with an adjusted mean difference of 2.8 (95% confidence interval 1.3–4.3, *p* <  0.001) and 0.6 (0.4–0.9, *p* <  0.001), respectively.

**Conclusions:**

Six-month resistance training was associated with improved muscle strength and physical performance in patients with MHD.

## Background

The number of patients with end-stage renal disease is increasing worldwide due to the increased prevalence of diabetic and hypertensive nephropathy with aging, resulting in an increased number of patients who receive maintenance hemodialysis (MHD) [1, 2]. Since MHD is associated with poor quality of life (QOL) and survival, the identification of risk factors and appropriate interventional strategies for improving QOL and outcomes have been intensively investigated [3–5]. Following this, reduced muscle strength and physical performance is considered to be one of the most important common prognostic indices of low QOL and poor survival in patients on MHD [3–5].

In contrast, it is well known that physical exercise has a positive impact on muscle strength and physical performance in healthy elderly patients, regardless of the type of exercise [6]. In addition, many studies have assessed the benefits of strength training from bench to bedside; these include anabolic changes in skeletal muscle messenger ribonucleic acid, or in muscle insulin-like growth factor-I protein, muscle mass, sleep quality, psychiatric status, and quality of life [7–10]. Among the several forms of exercise training, intra-dialytic resistance training has been suggested as a potential strategy to correct and/or prevent reductions of muscle strength and physical performance in MHD patients, considering its convenience, adherence, and safety, with little influence on cardiovascular systems [11, 12]. However, most studies evaluated the short-term impact on muscle strength or physical performance within 3 months by enrolling only a small number of patients (< 50), and little evidence is available regarding the association between resistance training during MHD and muscle strength or physical performance in a longer time period and with a larger number of patients [13–18]. The purpose of this multicenter retrospective observational study was to evaluate the association between the 6-month intra-dialytic resistance training and serial changes in muscle strength and physical performance.

## Methods

### Study patients

This retrospective observational study included a total of 306 patients on MHD. MHD was provided at outpatient dialysis units three times per week, and they underwent an intra-dialytic resistance exercise program for 6 months between April 2012 and July 2016 at 18 Kaikokai group hospitals in Japan. Thus, we only included patients who basically adhered to, and were compliant with the 6-month training programs; we can take from this that our study has a significant selection bias. The data of patients on MHD from these 18 hospitals had been gathered and recorded in the database at Nagoya Kyoritsu Hospital in a daily clinical practice and the study data presented in the tables were retrospectively extracted from this database as a secondary usage. The study protocol complied with the Helsinki Declaration standards and was approved by the Ethical Committee of Nagoya Kyoritsu Hospital (Approval No. K094–03). The requirement of written informed consent was waived as this study used retrospective data obtained from the aforementioned database. The patient’s right to avoid enrollment was pledged in an opt-out fashion. Extracted data included patient backgrounds, laboratory data, Kt/V as an index of hemodialysis adequacy (ideal value in the present study ≥1.4), normalized protein catabolic rate (n-PCR) as an index of nutritional status (ideal range in the present study 0.9–1.4 g/kg/day), and percent creatinine generation rate (%CGR) as an index of muscle mass compared to healthy individuals (ideal value in the present study ≥100%) [13–18]. Percent knee extension muscle power to dry body weight (pKEMP-dBW) was collected as representative values for the muscle strength of lower extremities. We evaluated physical performance using short physical performance battery (SPPB), where an SPPB ≤8 was defined as the presence of sarcopenia [19]. The authors had full access to the data and take responsibility for its integrity.

### Resistance training and measurement of muscle strength

**Fig. 1:**
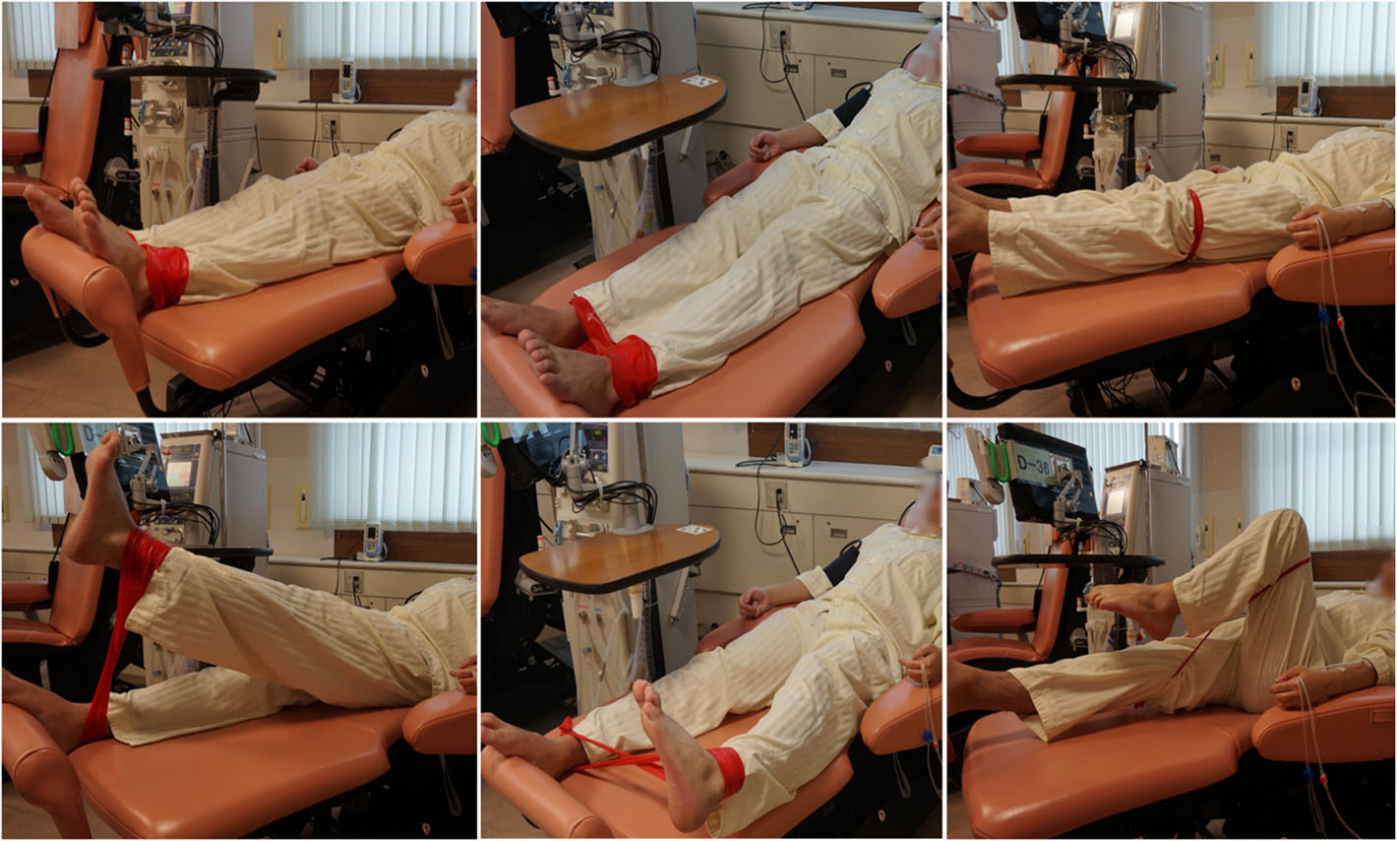
Protocol of Resistance Training. Resistance training consists of two sets of 10 repetitions for knee extension (Left), hip abduction (Middle), and hip flexion (Right) using an elastic band (TheraBand Resistance Band Loops, THERABAND, Ohio, USA), encircling both ankles or above the knees in a sitting or supine position, depending on patient preference or ability. Resistance training was performed with slow movement, taking 8 s to complete each resistance training

Resistance training was provided for 6 months, under the supervision of training therapists at first, then under a self-training manner during MHD three times per week on an outpatient basis. The training protocol consists of two sets of 10 repetitions of three types of resistance trainings of the lower extremities: knee extension, hip abduction, and hip flexion using an elastic band (TheraBand Resistance Band Loops, THERABAND, Ohio, USA) encircling both ankles or above the knees, in a sitting or supine position depending on patient preference or ability (Fig. 1). There were four colors of elastic bands and the resistance strength could be determined by the color. Resistance training was performed with slow movement, taking 8 s to complete each resistance training, consisting of 4 s eccentric and 4 s concentric movements. Hence, the total time of daily resistance training was 3 (kinds of training) * 10 (repetitions) * 2 (sets) * 8 s = 8 min; this took approximately 15 min per day to complete, including preparations and intervals. We employed slow resistance training in our daily clinical practice because slow resistance training can be performed at a lower intensity, with little influence on the cardiovascular system [20]. Resistance strength was set based on the Borg rating of perceived exertion and rating 13 (somewhat hard) was set as the targeted strength [21]. The strength was re-evaluated in detail every 12 weeks in our daily clinical practice, whereas the resistance strength was adjusted at each training session based on the Borg rating as mentioned above. Thus, by selecting a color of the elastic band patients could select the resistance strength by themselves. Adverse events were defined as clinical events that occurred during intra-dialytic resistance training and required any kind of therapeutic intervention. Knee extension muscle strength was measured using a dynamometer (μTas F-1 handheld dynamometer, Anima, Tokyo, Japan) at least twice before each MHD session, with the highest score being recorded. We used a handheld dynamometer because it was an easy to use, commercially available device widely used in Japan to measure knee strength [22, 23]. Although we could not source any validation data, the measurements were performed by an experienced therapist who was familiar with the device. Therefore, we believe that an accurate reading was obtained from this device. In addition, from a statistical viewpoint, because the comparison of serial changes was made using data from the same device, we do not expect the choice of device to affect the results.

### Endpoint and statistical analysis

Primary outcome measures were set as serial changes in the average pKEMP-dBW of the right and left legs and the SPPB between the baseline and 6 months. The average pKEMP-dBW was calculated by the following formula: (highest pKEMP-dBW in right leg + highest pKEMP-dBW in left leg)/2. Secondary outcome measures were set as serial changes in pKEMP-dBW in each leg and 6 min walk distance. All values were measured before MHD. Continuous variables were summarized using the mean ± standard deviation and categorical variables were summarized by percentages. The adjusted mean difference and 95% confidence interval (CI) in the primary and secondary outcome measures during the 6 months were estimated using a multivariate linear regression model adjusted for age and sex. The significance level for our statistical analysis was set at 0.05 with the two-sided alternative hypothesis. If the Bonferroni correction was applied to the significance level to consider the problem of multiple comparisons, readers can also set the significance level at 0.01. All statistical analyses were performed using the R software (version 3.4.2).

## Results

Patient backgrounds are shown in Table 1. The mean age was 70 ± 11 years, 52.3% of the patients were men, and the dry weight was 55.6 ± 11.3 kg with 159 ± 8 cm body height. About half of the patients (44.2%) reported that they had a habit of exercise. However, 24.4% patients were current smokers and 20.6% had a habit of alcohol consumption. Sarcopenia, defined as SPPB ≤8, was present in 21.4% patients, with a %CGR of 105 ± 28 at baseline. Their hemodialysis adequacy was acceptable, with a Kt/V of 1.65 ± 0.29, and their nutritional status was good, with an n-PCR of 0.89 ± 0.18 g/kg/day.

**Table 1:** Patient Backgrounds

Parameter	*n* = 306
Age, years	70 ± 11
Male	52.3%
Height, cm	159 ± 8
Weight	
Set dry weight, kg	55.6 ± 11.3
Pre-HD weight, kg	56.8 ± 11.5
Exercise habit	44.2%
Current smoker	24.4%
Alcohol consumption habit	20.6%
Laboratory data	
Creatinine, mg/dL	10.2 ± 2.6
Blood urea nitrogen, mg/dL	61.3 ± 15.7
Albumin, g/dL	3.6 ± 0.3
Hemoglobin, g/dL	11.0 ± 0.9
Total cholesterol, mg/dL	157 ± 33
Kt/V	1.65 ± 0.29
n-PCR, g/kg/day	0.89 ± 0.18
%CGR	105 ± 28
Sarcopenia	21.4%

Continuous variables were summarized using the mean ± standard deviation and categorical variables were summarized by percentages

*%CGR* percent creatinine generation rate, *HD* hemodialysis, *n-PCR* normalized protein catabolic rate

Regarding primary outcome measures shown in Table 2, both pKEMP-dBW and SPPB showed a slight but significant increase from 42.3 ± 14.9% to 45.7 ± 14.9% (adjusted mean difference, 2.8; 95% CI, 1.3–4.3, *p* <  0.001) and from 10.0 ± 2.6 to 10.4 ± 2.5 (0.6, 0.4–0.9, *p* <  0.001), respectively. In this study, the sample sizes required to detect the difference in primary outcome measures for muscle strength and physical performance were calculated to be 65 and 76, respectively, for a two-sided paired-t test with an alpha error of 0.05 and beta error of 0.80. Secondary outcome measures showed similar results to those of the primary outcome measures, where right and left pKEMP-dBW increased significantly after 6 months (Table 2). In contrast, there was no statistical change in the 6 min walk distance (*p* = 0.988). There were no adverse events during the study period.

**Table 2:** Serial Changes of Primary and Secondary Outcome Measures

Parameter	Baseline	6-Months Later	Difference (95% CI)^a^	*P*-value
Primary Outcome				
Average pKEMP-dBW, %	42.3 ± 14.9	45.7 ± 14.9	2.8 (1.3–4.3)	< 0.001
SPPB	10.0 ± 2.6	10.4 ± 2.5	0.6 (0.4–0.9)	< 0.001
Secondary Outcome				
Right pKEMP-dBW, %	42.6 ± 15.2	46.5 ± 15.7	3.6 (1.9–5.3)	< 0.001
Left pKEMP-dBW, %	41.9 ± 15.5	44.8 ± 15.0	2.1 (0.5–3.7)	0.012
Six minute walk distance, m	324 ± 175	317 ± 212	0 (−21–22)	0.988

Continuous variables were summarized using the mean ± standard deviation

^a^Adjusted for age and sex

*CI* confidence interval, *pKEMP-dBW* percent knee extension muscle power to dry body weight, *SPPB* short physical performance battery

## Discussion

In this multicenter retrospective observational study, we demonstrated that the 6-month resistance training program was associated with (1) increased muscle strength of the lower extremities, evaluated by pKEMP-dBW, and (2) increased physical performance, evaluated by SPPB in 306 patients with MHD. Considering that previous studies evaluated the impact of intra-dialytic resistance training from the data of a very limited number of patients (up to 79 before randomization, with the majority of studies providing a short-term 12-week training program), our data provide physicians with complementary insights in the resistance exercise training field for patients on MHD [13–18].

### Impact on muscle strength

As all previous studies reported the improvement of muscle strength of lower extremities attributable to 3 to 6 months intra-dialytic resistance training, regardless of its intensity, our results are consistent with these reports [13–18]. For example, Cheema B, et al. demonstrated a statistically significant 15.2% ± 15.4% improvement in total muscle strength associated with resistance training, whereas the control group showed a − 2.4% ± 13.8% change and no statistical significance [15]. This is consistent with our result of approximately 8% improvement in pKEMP-dBW. Since the improvement of muscle strength due to resistance training has also been reported in aging adults with or without diabetes, it is without a doubt that there is a favorable impact on muscle strength from resistance training [24–26]. In contrast to previous reports, there are two different points in the present study. That is, we provided resistance training in a self-training and slow movement manner. A recent randomized controlled trial (*n* = 296) of a 6-month personalized walking exercise program at home improved physical performance, including a 6-min walking test in patients on MHD [27]. Thus, we speculate that self-resistance training programs at home have the potential to be applicable for improving muscle strength in patients on MHD and this should be evaluated in a future study. In addition, slow resistance training could be performed at a lower intensity with little influence on cardiovascular systems, maintaining the anabolic effect on the muscles [20].

### Impact on physical performance

First of all, we employed SPPB, the 6-min walking test, and gait-speed as representative values describing patients’ physical performance in this discussion section [19]. By this definition, there is conflicting evidence present regarding the impact of resistance training on physical performance in patients on MHD [13–15, 17, 18]. For example, Headley S and Chen JL et al. reported the positive impact of resistance training on physical performance after 12 weeks of training in their interventional study, with data from 10 patients in 2002, and after presumably 18 weeks of training (36 sessions, twice a week), with data from 50 patients in 2010 [13, 17]. However, three other studies did not report a significant change in physical performance after 12 weeks of training [14, 15, 18]. One of the possible mechanisms of this discrepancy is the short training period. In fact, all negative results were reported by trials which provided 3-month training and we speculate that a long-term training period is needed as the anabolic effect on muscle fibers may need more time in elderly patients [28]. The other possible mechanism for the discrepancy is the low statistical power due to the small number of patients who receive intervention (*n* ≤ 25 in all negative studies) [14, 15, 18]. Consequently, two important aspects of the present study included the large number of study population (*n* = 306) and 6-month resistance training, providing physicians with clinically relevant insights. At any rate with our results, we speculate that resistance training has the potential to prevent the progression of sarcopenia and frailty in patients with MHD over a longer time period, improving QOL and survival. Further evaluation should be warranted for long-term efficacy.

### Clinical implication

Although our study was observational, and as such we could not assess the impact of resistance training accurately, our findings provide physicians with complementary insights in the field of resistance exercise training for patients on MHD. Given that our study included a large number of patients with a long follow-up time, we provide additional evidence and validation in support of previous reports describing the short term favorable impact of resistance training, or those examining a variety of exercise training programs in patients on MHD [7–18].

### Study limitations

Our study has several limitations that warrant mention. Firstly, we could not evaluate several risk factors that are associated with muscle wasting in patients with MHD, such as underlying disorders, hormonal alterations, inflammation, concurrent comorbidities or nutritional interventional information, due to the retrospective nature of this study [6, 12, 15]. However, in the present study, the nutritional status of the study population could be estimated by baseline n-PCR, albumin, or cholesterol levels (Table 1) [29, 30]. Secondly, we only included patients who had completed the 6-month training programs and our study has significant selection bias, although previous randomized controlled trials also had similar selection bias, with good adherence shown by the high completion rate of the study [13–18]. Third, we did not measure muscle mass in a daily clinical practice as previous studies have demonstrated that an improvement in muscle strength is attributable to the improvements in muscle mass [5, 24]. Thus, we could not collect data relating to muscle mass in this retrospective observational study. However, we did present data on the %CGR as an index of muscle mass. Finally, it is possible that some of the study patients self-trained at home, as 44.2% had an exercise habit at baseline. However, considering the increasing interest in obtaining real-world observational data in clinical decision making as complementary evidence for those from randomized controlled trials, we believe the importance of our results overweight these limitations [31, 32].

## Conclusions

Six-month resistance training was associated with improved muscle strength and physical performance in patients with MHD.

## Abbreviations

%CGR: Percent creatinine generation rate
HD: Hemodialysis
MHD: Maintenance hemodialysis
n-PCR: Normalized protein catabolic rate
pKEMP-dBW: Percent knee extension muscle power to dry body weight
QOL: Quality of life
SPPB: Short physical performance battery
